# The ascension of wildlife rabies: a cause for public health concern or intervention?

**DOI:** 10.3201/eid0104.950401

**Published:** 1995

**Authors:** C E Rupprecht, J S Smith, M Fekadu, J E Childs

**Affiliations:** Centers for Disease Control and Prevention, Atlanta, Georgia 30333, USA. cyr5@ciddvd1.em.cdc.gov

## Abstract

The epidemiology of rabies in the United States has changed substantially during the last half century, as the source of the disease has changed from domesticated animals to wildlife, principally raccoons, skunks, foxes, and bats. Moreover, the changes observed among affected wildlife populations have not occurred without human influence. Rather, human attraction to the recreational and economic resources provided by wildlife has contributed to the reemergence of rabies as a major zoonosis. Although human deaths caused by rabies have declined recently to an average of one or two per year, the estimated costs associated with the decrease in deaths amount to hundreds of millions of dollars annually. In future efforts to control rabies harbored by free-ranging animal reservoirs, public health professionals will have to apply imaginative, safe, and cost-effective solutions to this age-old malady in addition to using traditional measures.


### The Ascension of Wildlife Rabies: A Cause for Public Health Concern or Intervention?

**Charles E. Rupprecht, V.M.D., Ph.D., Jean S. Smith, M.S.**
**Makonnen Fekadu, D.V.M., Ph.D., and James E. Childs, Sc.D.**
Centers for Disease Control and Prevention, Atlanta, Georgia, USA

The epidemiology of rabies in the United States has changed substantially during the last half century, as the source of the disease has changed from domesticated animals to wildlife, principally raccoons, skunks, foxes, and bats. Moreover, the changes observed among affected wildlife populations have not occurred without human influence. Rather, human attraction to the recreational and economic resources provided by wildlife has contributed to the reemergence of rabies as a major zoonosis. Although human deaths caused by rabies have declined recently to an average of one or two per year, the estimated costs associated with the decrease in deaths amount to hundreds of millions of dollars annually. In future efforts to control rabies harbored by free-ranging animal reservoirs, public health professionals will have to apply imaginative, safe, and cost-effective solutions to this age-old malady in addition to using traditional measures.

Rabies virus is the type species (serotype 1) of the *Lyssavirus* genus, a group of morphologically similar, antigenically and genetically related, negative-stranded RNA viruses, with a near global distribution (1). The lyssaviruses (Table 1) are well adapted to particular mammalian species (2) and rarely initiate panzootics. The public health threat of rabies as a preeminent zoonosis relates to the acute, incurable encephalitis that results from transmission of the virus by the bite of an infected animal. An estimated 40,000 to 100,000 human deaths are caused by rabies each year worldwide; in addition, millions of persons, primarily in developing countries of the subtropical and tropical regions (3), undergo costly postexposure treatment (PET). Although the number of human rabies cases has been significantly reduced in the United States, the total number of animal rabies cases approached historical limits in 1993. To appreciate the public health significance that lyssaviruses continue to play as persistent and emerging infectious agents, one must understand certain human activities, such as recent animal translocations (i.e., the natural or purposeful change by humans of the normal home range or geographic distribution of an animal) and animal ecology.

Address for correspondence: Charles E. Rupprecht, Centers for Disease Control and Prevention, 1600 Clifton Rd., MS G33, Atlanta, GA 30333, USA; fax 404-639-1058; e-mail cyr5@ciddvd1.em.cdc.gov.

### Historical Perspectives

The history of rabies in the New World reflects the interaction of chance, evolutionary constraint, ecologic opportunism, and human surveillance activities. Rabies may have existed in the United States before European colonization and the introduction of domestic animals incubating the disease. Various pathogens could have migrated during the exchanges of fauna and human populations over the Bering Strait some 50,000 years ago; folklore of a rabies-like malady among native people throughout the Pacific Northwest supports this notion (4). Records at the time of the Spanish conquest in Middle America associate vampire bats with human illness (5). If chiropteran rabies viruses were present and well established in the New World at the time of continental interchange, terrestrial virus counterparts also could have been present. Nonetheless, the first indication of terrestrial rabies did not surface until 1703 in what is now California (5). Dog and fox rabies outbreaks, reported commonly in the mid-Atlantic colonies throughout the late 1700s (4), were probably exacerbated by the introduction of dogs and red foxes (*Vulpes vulpes*), imported for British-style fox hunting, throughout New England in the 1800s; fox rabies epizootics ensued and spread to the eastern United States by the 1940s to 1950s (5,6). Skunk rabies reports were also frequent throughout the western states by the 19th century, and they were replete with cowboy tales of “phobey cats” (5).

Table 1. Recognized members of the genus Lyssavirus, family *Rhabdoviridae*

| Lyssavirus | Reservoir | History |
| --- | --- | --- |
| Rabies | Found worldwide, except for a few island nations, Australia, and Antarctica. Endemic and sometimes epidemic in a wide variety of mammalian species, including wild and domestic canids, mustelids, viverrids, and insectivorous and hematophagous bats; >25,000 human cases/year, almost all in areas of uncontrolled domestic dog rabies. | Descriptions of clinical disease in Greek and Roman documents. In the late 1800s, Pasteur attenuated the virus by serial passage and desiccation to vaccinate humans and animals. Pathognomonic inclusions in nerve cells described by Negri in 1903. An immunofluorescence test for rabies viral antigen developed in the 1950s. |
| Lagosbat | Unknown, but probably fruit bats. 10 cases identified to date, including 3 in domestic animals, in Nigeria, South Africa, Zimbabwe, Central African Republic, Senegal, and Ethiopia. No known human deaths. | Isolated in 1956 from brain of Nigerian fruit bats (*Eidolon helvum*) at Lagos Island, Nigeria, but not characterized until 1970; 3 cases in domestic animals initially diagnosed as rabies, but weak immunofluorescence led to suspicion of "rabies-related" virus, later confirmed by typing with monoclonal antibodies or nucleotide sequence analysis. Marginal cross-protection with rabies vaccines. |
| Mokola | Unknown, but probably an insectivore or rodent species. Cases identified in Nigeria, South Africa, Cameroon, Zimbabwe, Central African Republic, and Ethiopia; 17 cases known, including 9 domestic animals and 2 human cases. | First isolated from *Crocidura* sp. shrews trapped in Mokola Forest near Ibadan, Nigeria, in 1968. Characterized in 1970. Like Lagos bat virus, evidence of infection with Mokola was recognized only by poor reaction with anti-rabies reagents. 7 domestic animal cases in Zimbabwe in 1981 and 1982 prompted serologic survey and identification of antibodies to Mokola in rodents, especially bushveld gerbils (*Tatera leucogaster*). No cross-protection with rabies vaccines. |

Although individual reports document a high incidence of dog rabies at the beginning of the last century, no national surveillance system existed. Human deaths from rabies in the United States were not commonly reported; the highest official record was of 143 cases, from a survey of death certificates in 1890. During 1938, when rabies in humans and other animals became a nationally reportable disease, the total number of rabies cases reported was 9,412 per year (mostly in domesticated species), with 47 human deaths. These numbers are certainly underestimates, since surveillance was limited, and sensitive diagnostic tests for human and animal rabies were not developed until the mid-1950s.

An epizootiologic transition began in the United States in the 1920s, when rabies prevention efforts were no longer focused exclusively on human vaccination but began to include programs for the control of rabies in dogs. Domestic animal cases gradually declined, largely as a result of local dog rabies control programs that included vaccination, stray animal removal, and leash and muzzle ordinances. However, as such cases decreased, surveillance systems designed to track the source of infection for residual domestic animal foci detected increased cases in wild species. By 1960, rabies was diagnosed more frequently among wildlife than among domesticated animals. In 1971, rabies was reported for the first time from all 48 contiguous states and Alaska. Skunks (primarily the striped skunk, *Mephitis mephitis*) formed the major animal reservoir from 1961 to 1989, until they were unexpectedly supplanted by the raccoon (*Procyon lotor*) during the rabies outbreak in the mid-Atlantic and northeastern states (7). This epizootic is believed to have started during the late 1970s by the

Table 1. (continued)

| Lyssavirus | Reservoir | History |
|---|---|---|
| Duvenhage | Unknown, but probably insectivorous bats. Cases identified in South Africa, Zimbabwe, and Senegal; 4 cases known, including 1 human death. No cases in domestic animals. | First identified in 1970 in rabies-like encephalitis in man bitten by an insectivorous bat near Pretoria, South Africa. Virus named after the victim. Although Negri bodies detected in histologic examination of brain tissue, negative immunofluorescence tests led to suspicion of rabies-related virus, subsequently confirmed by antigenic and genetic typing. Marginal cross-protection with rabies vaccines. |
| European bat Lyssavirus 1 (EBLV1) | European insectivorous bats (probably *Eptesicus serotinus*); >400 cases in bats. 1 confirmed human case in 1985 and a suspect case in 1977. No known domestic animal cases. | Although cases in European bats were reported as early as 1954, identification of the virus was not attempted until 1985, when the first of 100 infected bats was reported in Denmark and Germany. Almost all cases are in the common European house bat, *E serotinus*. Marginal cross-protection with rabies vaccines. |
| European bat Lyssavirus 2 (EBLV2) | European insectivorous bats (probably *Myotis dasycneme*); 5 cases identified, including 1 human death. No known domestic animal cases. | First identified in isolate from Swiss bat biologist who died of rabies in Finland. Marginal cross-protection with rabies vaccines. |

translocation of infected animals from a southeastern focus of the disease.

The epidemiology of human rabies has also changed considerably over the last 50 years (8,9). From 1946 to 1965, 70% to 80% of human rabies cases occurred after a known exposure (most often a dog bite), and 50% of the cases before 1975 occurred after treatment with suboptimal vaccines. Over the last decade, 80% of rabies-related human deaths were among persons who had no definitive history of an animal bite (Table 2), and none resulted from postexposure prophylaxis failures. Almost all the recent human cases occurred after an animal exposure that was unrecognized by the patient as carrying a risk for rabies infection. The apparent source of human rabies has also changed: 14 of the 18 cases acquired in the United States since 1980 involved rabies variants associated with insectivorous bats (10).

The latest report, in March 1995, typifies recent trends. A bat, subsequently found to be rabid, was found in the bedroom of a 4-year-old girl in Washington State. The child denied any contact with the bat, and no postexposure treatment was initiated. A bat-associated rabies virus variant was later identified in biopsy specimens from the

Table 2. Human rabies cases in the United States by exposure category, 1946-1995*

| | Exposure source | | | | Case |
|---|---|---|---|---|---|
| Years | Domestic | Wildlife | Other | Unknown (%) | total |
| 1946-1955 | 86 | 8 | 0 | 26 (22) | 120 |
| 1956-1965 | 21 | 7 | 0 | 10 (26) | 38 |
| 1966-1975 | 6 | 7 | 1 | 2 (13) | 16 |
| 1976-1985 | 6 | 1 | 2 | 11 (55) | 20 |
| 1986-1995* | 2 | 2 | 0 | 14 (78) | 18 |

* Through Oct. 1995.

child and from the bat's carcass (11). Despite the current prominence of raccoons as the largest wildlife reservoir in the United States (12), no documented human rabies cases have been associated with this ubiquitous carnivore.

### The Cost of Prevention

Rabies prevention and control strategies in the United States have succeeded in lowering the number of human rabies deaths to an average of one to two per year. However, the reason for this low mortality level is a prevention program estimated to cost $230 million to $1 billion per year (13-15). This cost is shared by the private sector (primarily the vaccination of companion animals) and by the public (through animal control programs, maintenance of rabies laboratories, and subsidizing of rabies PET).

Accurate estimates of these expenditures are not available. The number of PETs given annually in the United States is unknown, although the total must be substantially greater than the minimum of 20,000 estimated in 1980 to 1981 (16) when vaccine distribution was more tightly regulated. As rabies becomes epizootic or enzootic in a region, the number of PETs increases (17). Although the cost varies, a course of rabies immunoglobulin and five doses of vaccine given over a 4-week period typically exceeds $1,000. Potential exposure to a single rabid kitten in New Hampshire recently led to the treatment of more than 650 persons at an estimated cost of $1.5 million (18). Surveillance-related costs also rise as rabies becomes entrenched in wildlife. During 1993, the New York State rabies diagnostic laboratory received approximately 12,000 suspected animal submissions. This compares with approximately 3,000 submissions in 1989, before raccoon rabies became epizootic. In New Jersey, rabies prevention expenditures in two counties increased from $768,488 in 1988, before the raccoon epizootic, to $1,952,014 in 1990, the first full year of the epizootic (15); vaccination of pet animals accounted for 82% of this total. Vaccinated domestic animals are normally administered a booster vaccine dose after a known or suspected rabid animal exposure (19). This further increases costs, as wildlife rabies epizootics escalate. The cost per human life saved from rabies ranges from approximately $10,000 to $100 million, depending on the nature of the exposure and the probability of rabies in a region (20).

What's more, most economic analyses do not take into account the psychological trauma caused by human exposure to rabies, the subsequent euthanasia of pets, or the loss of wildlife resources during rabies outbreaks. Rabies in wildlife has now reached historically high levels in the United States (12), and the costs of preventing human rabies are mounting.

### Human Influences and the Role of Translocation

The colonization of the New World had a profound effect upon native fauna and consequent rabies epizootiology. Large-bodied carnivores, such as bears, cougars, wolves, and wolverines, were perceived as dangerous and killed outright. A few Carnivora have persisted and flourished. For example, the coyote (*Canis latrans*), a highly adaptable canid and the subject of many unsuccessful control programs, has been gradually expanding its range northward and eastward. Despite their widespread distribution and abundance (even in suburban neighborhoods), rabid coyotes have been reported rarely and sporadically, except for a brief period from 1915 to 1917, when an extensive outbreak occurred in portions of Utah, Nevada, California, and Oregon.

While dog rabies has been largely controlled, a region of southern Texas that borders Mexico has persisted as a focus of both dog and coyote rabies. The number of cases of coyote rabies has gradually risen in this area since the late 1980s, accounting for 46 of the 50 cases of coyote rabies reported in the United States during 1991, 70 of 75 cases in 1992, and 71 of 74 cases in 1993 (12). The outbreak of coyote rabies has spread to the vicinity of San Antonio. One of the dangers of this outbreak is the continued spillover into the domestic dog population (21); at least 25 rabid dogs were reported from the area in 1991, 41 in 1992, and 54 in 1993 (12). Human rabies closely parallels the disease in domestic animals; at least two human deaths (in 1991 and 1994), probably due to coyote-dog interactions, have been associated with this canid outbreak in Texas (10,22).

The translocation of infected coyotes from the south Texas focus is believed to be responsible for the transmission of this rabies variant to dogs in at least two other states: a single hunting dog in Alabama during 1993 (12) and at least seven cases of apparent dog-to-dog rabies transmission in Florida in 1994 (21). Expanded surveillance similar to that done in 1977 with the raccoon rabies focus in the mid-Atlantic region (7) is warranted for this canid virus. In this effort, state health departments should monitor unusual occurrences (such as the increased submission of canid specimens to the diagnostic facility), tracking of their time and location, and establishment of suitable public health interventions. These would include restricting further animal movements and enforcing mandatory companion animal rabies vaccination. Assessing control efforts is an important component of any intervention. In addition to the problems posed by the emergence of the coyote as a reservoir for rabies, the potential translocation of other species should be recognized.

Since the transmission of rabies by a bat was first reported in 1953, rabid insectivorous bats have caused an average of 700 to 800 cases annually, and have been found throughout the United States, excluding Alaska and Hawaii (12). The discovery of these cases, coincident with the marked reduction of canine rabies cases, has afforded a certain epidemiologic luxury to enhance surveillance among wildlife. Similar to the Carnivora, the chiropteran families most important in rabies perpetuation (e.g., *Vespertilionidae, Molossidae*) have several species that are highly adaptable, abundant, and widespread. Rabies virus variants maintained by insectivorous bats appear to be exchanged largely independently from those in terrestrial mammalian reservoirs (23), despite documented spillovers. A similar epidemiologic situation exists among European bats, but with *Lyssavirus* genotypes (24) that can be readily differentiated from New World rabies isolates. The role of bats in Africa (25,26) in *Lyssavirus* maintenance is less clear (Table 1). Infections with non-rabies lyssaviruses have resulted in rabies vaccine failures (27). Such infections raise the specter of potentially serious public health consequences if introduced and subsequently established in susceptible bat populations. How probable is this scenario?

The distances between Africa, Eurasia, Pacific Oceania, and the New World mitigate against the dispersal, migration, and introduction of healthy bats without human intervention (28). However, several recent events illustrate the opportunity for the transoceanic transfer of rabies-infected bats. In March 1986, researchers from Canada inadvertently shipped a big brown bat (*Eptesicus fuscus*) that was incubating rabies virus to colleagues in Tubingen, Germany. When the bat became ill and was euthanized, a diagnosis of rabies was made (29). A similar event occurred when Boston researchers collected a dozen wild big brown bats from Massachusetts during July 1994 and exported them to researchers in Denmark. By December 1994, six of the imported bats had died and were confirmed as positive for rabies virus by the Danish Veterinary Services, State Institute for Virus Research (L. Miller, pers. comm.).

Commercial enterprises also serve as vehicles for the accidental translocation of animals infected with rabies virus. The first confirmed nonindigenous case of rabies in Hawaii resulted from the accidental introduction of a big brown bat (30). In March 1991, a bat was captured within a transport container unloaded from a ship in Honolulu harbor. The container held automobiles from Michigan loaded into the container ship in California. The local department of health laboratory diagnosed rabies; this was later confirmed, and the virus was characterized antigenically at the Centers for Disease Control and Prevention. The strain was a variant common to *E. fuscus* in the midwestern and western United States. None of the three instances cited above appear to have resulted in secondary cases or establishment of the virus in foreign animal populations.

No unintended importations of non-rabies lyssaviruses to the United States have been documented. The likelihood of accidental introduction, escape, survival, and perpetuation of infected exotic bat species into the United States is remote. However, other more recent deliberate translocation activities may significantly enhance the probability of such introductions.

During 1994, a number of improperly issued federal permits allowed as many as several thousand wild bats to be imported to the United States for sale in the commercial pet trade. These animals were primarily Egyptian tomb bats (*Rousettus aegyptiacus*), although several other bat species were imported as well. Sales of imported bats (and their offspring) to private collectors or as pets in the United States are prohibited, according to the Foreign Quarantine Regulations (42 CFR 71.54). Animals that may be vectors of diseases of public health concern are eligible for entrance only for restricted uses at accredited zoos or research institutions, where contact with the general public is limited. Imported bats that will be legally displayed normally undergo an extended quarantine period.

Although no reports of lyssaviruses isolated from Egyptian fruit bats exist, active surveillance for such viruses has not been conducted. These bats are relatively common and widespread throughout the area that extends from Turkey and Cyprus to Pakistan, the Arabian peninsula, Egypt, and most of sub-Saharan Africa (31). Because they may roost by the thousands in a wide variety of habitats, there is ample opportunity for interaction with other Chiroptera, such as the widely distributed straw-colored (*Eidolon helvum*) or epauletted (*Epomophorus wahlbergi*) fruit bats; both of these species have been implicated in *Lyssavirus* epizootiology in Africa (25,26). The adaptability of Egyptian fruit bats should be a cause for concern because of the potential for survival and interaction among indigenous bat fauna, particularly in the southern United States. Additionally, beyond the obvious public health risks and foreign animal disease introduction, imported bat species should not be released into the wild because they may cause serious harm to local agriculture and may displace native species.

Bats serve many critical ecologic functions worldwide and generally avoid contact with humans. However, they may be infected with many pathogens without demonstrating obvious clinical signs of infection. When bats are placed in a private household or pet shop, the hazard of disease transmission to humans is greatly increased. Persons currently possessing imported bats should be advised not to display them in settings where human contact can occur.

### Intervention

Widespread, sustained population reduction of mammalian reservoirs to eliminate rabies is not justified (32) for ecologic, economic, and ethical reasons. Given the multispecies complexity and considerable geographic areas affected by wildlife rabies, and the opportunities for translocation, what alternative preventive strategies exist? Recent progress in implementing terrestrial wildlife rabies control programs elsewhere in the world has public health relevance for the United States. Oral rabies vaccination of the red fox with vaccine-laden baits is an integral aspect of rabies control throughout southeastern Canada and Europe, where more than 75 million doses of vaccine have been distributed over 5 million $km^2$ during the past two decades (33). Consequently, rabies incidence among wild and domestic animals has fallen, as have PETS for human rabies.

The raccoon rabies epizootic in the eastern United States provided renewed impetus for reconsidering oral vaccination technology, first conceived at the Center for Disease Control in the 1960s (34). The shift of the vaccination and baiting methods from a fox model to the raccoon involved extensive field and laboratory research during the 1980s. The existing attenuated rabies vaccines for foxes were shown to be less effective for raccoons and other carnivores (35,36). Additionally, studies of new candidate vaccines raised safety issues regarding vaccine-induced disease in wildlife (36).

In 1983, a vaccinia-rabies glycoprotein (V-RG) recombinant virus vaccine was developed (37) that has proven to be an effective oral immunogen in raccoons and various other important reservoir species (38); vaccine advantages include improved thermostability and an inability to cause rabies. (Only the gene for the surface glycoprotein of a vaccine strain of rabies virus was included in the recombinant virus.) When vaccine-laden baits are offered under natural conditions, contact with them by nontarget wildlife species cannot be totally excluded. However, studies of V-RG virus have shown no vaccine-associated morbidity, mortality, or gross pathologic lesions in more than 40 warm-blooded vertebrate species examined. Moreover, with rare exceptions, there has been no contact-transfer of vaccine between vaccinated and control animals housed together (38); viral recovery has been limited to a few anatomical sites over a 48-h interval (39).

While laboratory evaluations of target and nontarget species proceeded during 1987 to 1989, small-scale trials of V-RG were conducted in Belgium and France, with promising results (40). The first North American V-RG vaccine field trial began on August 20, 1990, on Parramore Island off the eastern shore of Virginia (41,42). This limited field trial demonstrated vaccine safety. Efficacy was also suggested: more than 80% of field-vaccinated raccoons survived a severe laboratory rabies challenge (7 months after V-RG release) to which more than 90% of control raccoons succumbed (43).

A 1991 Pennsylvania study site closely approximated the ecologic communities of the eastern United States targeted for use of V-RG

vaccine, while still maintaining relative biosecurity through its geographic barriers. The study at this site evaluated the rate of vaccine-laden bait contact and potential vaccine-related adverse effects among nonraccoon species, including rodents, carnivores, insectivores, and opossum. Raccoons and other furbearers demonstrated no adverse effects associated with vaccine contact. Examination of more than 750 nontarget individuals, representing 35 species, failed to demonstrate gross lesions suggestive of V-RG contact.

After these safety trials, the first efficacy field experiments began in New Jersey during 1992 (44). Between spring 1992 and autumn 1994, more than 100,000 vaccine-laden fishmeal polymer baits were distributed by hand and helicopter over an area of 56,000 hectares. This trial attempted to create a population of immunized raccoons across the northern Cape May Peninsula to prevent the spread of epizootic raccoon rabies from affected portions of the state. Surveillance demonstrated a significant decrease in the rate of spread and overall rabies incidence in the target and other monitored areas (44), suggesting the potential effectiveness of this strategy.

In the United States, oral vaccination of raccoons is now under way in Massachusetts (45), New York (46), and Florida, and an experimental extension of the program to coyotes is under way in south Texas. However, the future of such vaccination for wildlife in the United States may be seriously questioned. For oral vaccination to become an adjunct to traditional methods, the following major questions need to be answered: 1) What is the relationship between animal population density and the minimum density of vaccine/baits needed? 2) What level of herd immunity is necessary to eliminate rabies under various environmental circumstances? 3) What bait distribution techniques are optimal? 4) How can these methods be generalized from foxes and raccoons to other species, such as skunks, mongooses and dogs? 5) What long-term funding sources are available? 6) What are the various costs of rabies control and prevention methods? Given the problems inherent in wildlife control, the greater issue of extending these methods to the control of dog rabies in the developing world will be a challenge well into the next century.

*Dr. Rupprecht is chief of the Rabies Section, Ms. Smith is a research microbiologist, Dr. Fekadu is a research veterinary medical officer, and Dr. Childs is chief of the Epidemiology Section, Viral and Rickettsial Zoonoses Branch, Division of Viral and Rickettsial Diseases, National Center for Infectious Diseases, Centers for Disease Control and Prevention, Atlanta, Georgia.*

### Acknowledgment

The authors gratefully acknowledge the technical expertise of the staff of the Rabies and Epidemiology Sections, Viral and Rickettsial Zoonoses Branch, Division of Viral and Rickettsial Diseases, National Center for Infectious Diseases, Centers for Disease Control and Prevention, without whose assistance this work would not have been possible.

### References

1. World Health Organization. World survey of rabies 28 for the year 1992. Geneva: World Health Organization, 1994.
2. Wandeler A, Nadin-Davis SA, Tinline RR, Rupprecht CE. Rabies epizootiology: an ecological and evolutionary perspective. In: Rupprecht CE, Dietzschold B, Koprowski H, editors. Lyssaviruses. New York: Springer-Verlag, 1994:297-324.
3. Meslin FX, Fishbein DB, Matter HC. Rationale and prospects for rabies elimination in developing countries. In: Rupprecht CE, Dietzschold B, Koprowski H, editors. Lyssaviruses. New York: Springer-Verlag, 1994:1-26.
4. Winkler WG. Fox rabies. In: Baer GM, editor. The natural history of rabies. 1st ed. New York: Academic Press, 1975:3-22.
5. Baer GM. Rabies—an historical perspective. Infectious Agents and Disease 1994;3:168-80.
6. Carey AB, Giles RH, McLean RG. The landscape epidemiology of rabies in Virginia. Am J Trop Med Hyg 1978;27:573-80.
7. Rupprecht CE, Smith JS. Raccoon rabies—the re-emergence of an epizootic in a densely populated area. Semin Virol 1994;5:155-64.
8. Held JR, Tierkel ES, Steele JH. Rabies in man and animals in the United States, 1946-65. Public Health Rep 1967;82:1009-18.
9. Anderson LJ, Nicholson KG, Tauxe RV, Winkler WG. Human rabies in the United States, 1960 to 1979: epidemiology, diagnosis, and prevention. Ann Intern Med 1984;100:728-35.
10. Centers for Disease Control and Prevention. Human rabies—Alabama, Tennessee, and Texas, 1994. MMWR 1995;44:269-72.
11. Centers for Disease Control and Prevention. Human rabies—Washington state, 1995. MMWR 1995; 44:625-7.

12. Krebs JW, Strine TW, Smith JS, Rupprecht CE, Childs JE. Rabies surveillance in the United States during 1993. J Am Vet Med Assoc 1994;205:1695-709.
13. Stehr-Green JK, Schantz PM. The impact of zoonotic diseases transmitted by pets on human health and the economy. Vet Clin North Am Small Anim Pract 1987;17:1-15.
14. Fishbein DB, Arcangeli S. Rabies prevention in primary care: a four-step approach. Postgrad Med 1987;82:83-90.
15. Uhaa IJ, Dato VM, Sorhage FE, et al. Benefits and costs of using an orally absorbed vaccine to control rabies in raccoons. J Am Vet Med Assoc 1992;201:1873-82.
16. Helmick CG. The epidemiology of human rabies postexposure prophylaxis, 1980-1981. JAMA 1983;250:1990-6.
17. Centers for Disease Control and Prevention. Raccoon rabies epizootic: United States, 1993. MMWR 1994;43:269-73.
18. Centers for Disease Control and Prevention. Mass treatment of humans exposed to rabies—New Hampshire, 1994. MMWR 1995;44:483-6.
19. Centers for Disease Control and Prevention. Compendium of animal rabies control, 1995. MMWR 1995;44:(RR-2):1-9.
20. Fishbein DB, Robinson LE. Rabies. N Engl J Med 1993;329:1632-8.
21. Centers for Disease Control and Prevention. Translocation of coyote rabies—Florida, 1994. MMWR 1995;44:580-1, 7.
22. Centers for Disease Control and Prevention. Human rabies—Texas, Arkansas, and Georgia, 1991. MMWR 1991;40:765-9.
23. Smith JS, Orciari LA, Yager PA. Molecular epidemiology of rabies in the United States. Semin Virol (in press).
24. Bourhy H, Kissi B, Lafon M, Sacramento D, Tordo N. Antigenic and molecular characterization of bat rabies virus in Europe. J Clin Microbiol 1992;30:2419-26.
25. Swanepoel R, Barnard BJH, Meredith CD, et al. Rabies in southern Africa. Onderstepoort J Vet Res 1993;60:325-46.
26. King AA, Meredith CD, Thomson GR. The biology of southern Africa lyssavirus variants. In: Rupprecht CE, Dietzschold B, Koprowski H, editors. Lyssaviruses. New York: Springer-Verlag, 1994:267-96.
27. Foggin CM. Mokola virus infection in cats and a dog in Zimbabwe. Vet Rec 1983;113:115.
28. Wiles GJ, Hill JE. Accidental aircraft transport of a bat to Guam. J Mamm (full title) 1986;67:600-1.
29. World Health Organization Collaborating Center for Rabies Surveillance and Research. Bat rabies cases in the Federal Republic of Germany. World Health Organization Rabies Bulletin Europe 1986;10:8-9.
30. Sasaki DM, Middleton CR, Sawa TR, Christensen CC, Kobayashi GY. Rabid bat diagnosed in Hawaii. Hawaii Med J 1992;51:181-5.
31. Nowak RM. Walkers mammals of the world. 5th ed. Baltimore: Johns Hopkins University Press, 1991:198.
32. Debbie JG. Rabies control of terrestrial wildlife by population reduction. In: Baer GM, editor. The natural history of rabies. 2nd ed. Boca Raton, FL: CRC Press, 1991:477-84.
33. World Health Organization. Oral immunization of foxes in Europe in 1994. Wkly Epidemiol Rec 1995;70:89-91.
34. Baer GM. Oral rabies vaccination: an overview. Rev Infect Dis 1988;10 (Suppl 4):S644-8.
35. Rupprecht CE, Dietzschold B, Cox JH, Schneider L. Oral vaccination of raccoons (*Procyon lotor*) with an attenuated (SAD-B19) rabies virus vaccine. J Wildl Dis 1989;25:548-54.
36. Rupprecht CE, Charlton KM, Artois M, et al. Ineffectiveness and comparative pathogenicity of attenuated rabies virus vaccines for the striped skunk (*Mephitis mephitis*). J Wildl Dis 1990;26:99-102.
37. Wiktor TJ, Macfarlan RI, Reagan KJ, et al. Protection from rabies by a vaccinia virus recombinant containing the rabies virus glycoprotein gene. Proc Natl Acad Sci USA 1984;81:7194-8.
38. Rupprecht CE, Hanlon CA, Hamir AN, Koprowski H. Oral wildlife rabies vaccination: development of a recombinant virus vaccine. Transactions of the North American Wildlife Natural Resources Conference 1992;57:439-52.
39. Rupprecht CE, Hamir AN, Johnston DH, Koprowski H. Efficacy of a vaccinia-rabies glycoprotein recombinant virus vaccine in raccoons (*Procyon lotor*). Rev Infect Dis 1988;10 (4 Suppl):S803-9.
40. Aubert MFA, Masson E, Artois M, Barrat J. Oral wildlife rabies vaccination field trials in Europe, with recent emphasis on France. In: Rupprecht CE, Dietzschold B, Koprowski H, editors. Lyssaviruses. New York: Springer-Verlag, 1995:219-44.
41. Hanlon CA, Hayes DE, Hamir AN, et al. Proposed field evaluation of a rabies recombinant vaccine for raccoons *Procyon lotor*: site selection target species characteristics and placebo baiting trials. J Wildl Dis 1989;4:555-67.
42. Hanlon CA, Buchanan JR, Nelson E, et al. A vaccinia-vectored rabies vaccine field trial:ante- and post-mortem biomarkers. Rev Sci Tech 1993;99-107.
43. Rupprecht CE, Hanlon CA, Niezgoda M, Buchanan JR, Diehl D, Koprowski H. Recombinant rabies vaccines: efficacy assessment in free-ranging animals. Onderstepoort J Vet Res 1993;60:463-8.
44. Roscoe DE, Holste W, Niezgoda M, Rupprecht CE. Efficacy of the V-RG oral rabies vaccine in blocking epizootic raccoon rabies. Presented at the 5th Annual International Meeting of Rabies in the Americas, Niagara Falls, Ontario, Canada, 1994, Abstract, p.33.
45. Robbins AH, Niezgoda M, Levine S, et al. Oral rabies vaccination of raccoons (*Procyon lotor*) on the Cape Cod Isthmus, Massachusetts. Presented at the 5th Annual International Meeting of Rabies in the Americas, Niagara Falls, Ontario, Canada, 1994; Abstract, p. 29.
46. Hanlon CA, Trimarchi C, Harris-Valente K, Debbie JG. Raccoon rabies in New York State: epizootiology, economics, and control. Presented at the 5th Annual International Meeting of Rabies in the Americas, Niagara Falls, Ontario, Canada, 1994; Abstract, p.16.
